# Molecular Detection of Human Cytomegalovirus (HCMV) Among Infants with Congenital Anomalies in Khartoum State, Sudan

**DOI:** 10.2174/1874357901509010038

**Published:** 2015-12-10

**Authors:** Maha G. Ebrahim, Aisha S. Ali, Mohamed O. Mustafa, Dalal F. Musa, Abdel Rahim M. El Hussein, Isam M. Elkhidir, Khalid A. Enan

**Affiliations:** 1 Department of Microbiology, Faculty of Medical Laboratory Sciences, Al Neelain University, Khartoum, Sudan; 2 Department of Microbiology, Faculty of Medical Laboratory Sciences, National University, Sudan; 3 Departmemt of Virology, Central Laboratory, Ministry of Science and Communication, Khartoum, Sudan; 4 Department of Microbiology, Faculty of Medical Laboratory Sciences, Sudan University Science and Technology, Sudan; 5 Department of Microbiology and Parasitology, Faculty of Medicine, University of Khartoum, Khartoum, Sudan

**Keywords:** Congenital anomalies, ELISA, HCMV, PCR, Sudan

## Abstract

Human Cytomegalovirus (HCMV) infection still represents the most common potentially serious viral complication in humans and is a major cause of congenital anomalies in infants. This study is aimed to detect HCMV in infants with congenital anomalies.

Study subjects consisted of infants born with neural tube defect, hydrocephalus and microcephaly.

Fifty serum specimens (20 males, 30 females) were collected from different hospitals in Khartoum State. The sera were investigated for cytomegalovirus specific immunoglobin M (IgM) antibodies using enzyme-linked immunosorbent assay (ELISA), and for Cytomegalovirus DNA using polymerase chain reaction (PCR).

Out of the 50 sera tested, one patient’s (2%) sample showed HCMV IgM, but with no detectable DNA, other 4(8.2 %) sera were positive for HCMV DNA but with no detectable IgM.

Various diagnostic techniques should be considered to evaluate HCMV disease and routine screening for HCMV should be introduced for pregnant women in this setting. It is vital to initiate further research work with many samples from different area to assess prevalence and characterize HCMV and evaluate its maternal health implications.

## INTRODUCTION

Human Cytomegalovirus (HCMV) belongs to the Herpesviridae family and is a common cause of infection among humans. The virus undergoes periods of activity and periods of latency and, once a person has been infected, the virus remains in the host’s body indefinitely and can be reactivated due to many stress factors [1].

The virus is transmitted to fetuses *via* the transplacental route. The earlier the virus is transmitted to the fetus, the worst the prognosis and the greater the chance of severe malformations. The maternal infection can either be primary (in women who had never been infected before) or recurrent (by viral reactivation or reinfection by other viral strains) [2]. 

Congenital HCMV is a neglected problem worldwide, both at the health care workers level and the general public level. This is being attributed to the fact that most of the infections are asymptomatic and may not be recognized at birth. Moreover sequelae of HCMV are sometimes delayed in onset with the result that retrospective diagnosis becomes more challenging.

Finally, the wrong perception that congenitally infected infants (babies) born to seropositive mothers usually have normal outcome has led to less attention to congenital HCMV in developing countries [3]. However, it is becoming clear that in developing countries with high seropositive populations a high rate of HCMV infection and higher rate of congenital hearing loss in developing countries do exist [3, 4].

Prevention and control of HCMV remains an ongoing challenge. The high diversity and infection with multiple differing strain of the virus represent a potential barrier to the development of effective vaccines [5, 6]. Moreover, at the population level, there is certainly potential that vaccination of women with preexisting immunity might boost their immunity and reduce the incidence of congenital infection and/or disease in this population. Updated estimates of the produced congenital CMV and its impact are needed for increased awareness of congenital CMV infection and disease, allocation of public health resources, and determination of the cost-effectiveness of potential interventions [7, 8]. The current study aims to collect preliminary information regarding this problem in Sudan and highlight its extent and to stimulate further research to in this field in the country. 

## MATERIAL AND METHOD

**Study Site:** This is a descriptive cross-sectional neonates study conducted for 50 newborn infants with suspected HCMV infection (complained from neural tube defect, hydrocephalus and microcephaly). The study was conducted in the neonate intensive care units (NICUs) of three Hospitals in Khartoum State (Ibrahim Malik teaching Hospital, Khartoum Teaching Hospital and Omdurman Maternity Hospital), during September 2014 to January 2015.

**Samples Collection: **Blood samples were collected from infants with various congenital anomalies (n=50) after birth (age range was from one day to four months). Blood (2-5 ml) was collected in plain containers and transported on wet ice to the laboratory for immediate processing. Sera were separated from blood samples by centrifugation and stored at -20 till tested.

**Capture** **ELISA IgM: **Serum samples were tested for the presence of CMV IgM antibodies using 3^rd^ generation commercially available ELISA kits (GENESIS Diagnostic, Omega Diagnostic Group PLC, Cambridge Shine, UK), according to the manufacturer’s instructions.

**DNA Extraction: **Serum samples were processed by using commercial DNA extraction kit (Bio Basic Inc One Tube Viral DNA Isolation Buffer), which was performed according to the manufacturer’s instructions.

**Fig. (1):**
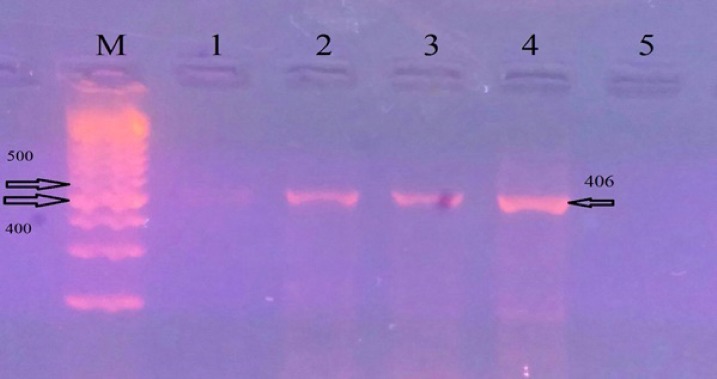
HCMV PCR results (406 bp) on 2 % agarose gel. Lanes 1-3 show positive patients Samples; Lane 4 shows positive control; Lane 5 shows negative control result of HCMV; M: 100 bp DNA size marker.

**DNA Amplification:** The outer prime sequences (Forward: 5-GGA TCC GCA TGG CAT TCA CGT ATG T-3, reverse: 5-GAA TTC AGT GGA TAA CCT GCG GCG A-3) were selected from a conserved region of the fourth exon of the HCMV immediate early (IE) gene, located in the Hind III-X fragment of the AD-169 strain [9, 10]. Optimized PCR reaction for HCMV DNA amplification was performed according to [10] briefly, 3ul of DNA extract was added to PCR premix (Maxime PCR premix kit (i-Tag)) containing i-Tag TM DNA polymerase, dNTP mixture and reaction buffer. Two ul of primers and 13ul of distilled water were then added to PCR premix. The PCR program consisted of 95° C for10 min, followed by 35 cycles of 95° C 45 s, 58° C 50 s and 72° C 45 s with a final extension at 72°C 5 min. The PCR was carried out in a total volume of 25ul and the amplified PCR product was detected by agarose gel electrophoresis. The product was visualized by staining with 0.15% Ethidium bromide using UV gel documentation system INGeNius. The expected size of immediate early (IE) gene amplicon was 406 bp (Fig. **1**).

### Ethical Review

The study was approved by the Ethical Review Committee (ERC) of the Ministry of Health Khartoum State, Sudan. Informed consents were obtained from parents or legal guardians of children.

## RESULTS 

A total of 50 newborn patients were enrolled in this study, 20 (40%) of them were males and 30 (60%) were females.

One male (2%) out of 50 neonates with microcephaly was found to be HCMV- IgM antibodies positive and 49 (98%) were found to be HCMV IgM antibodies negative.

In addition, 4(8.2%) samples were HCMV DNA positive. These samples were from 2 males and 2 female neonates all of them suffered from congenital neural tube defect and hydrocephalus). None of the microcephally patients were DNA positive. Fifty samples were used for comparison of both ELISA and PCR results, out of them, 4 samples were positive for PCR, one sample for both ELISA IgM and PCR and 46 samples were negative by both testes. This revealed an overall agreement of 94% (47/50) between the two tests Table **1**.

## DISCUSSION

Congenital CMV infection is the most common congenital infection worldwide. The rate of infection varies in different population and different age groups. The maternal primary or reactivated infection during pregnancy can lead to Congenital CMV infection, but maternal reinfection with different strains of CMV can rarely lead to congenital symptomatic infection. 

Although HCMV is a major public health problem throughout the world, only limited information is available about the incidence and the history of this infection in Sudan.

The aim of the present study was to determine prevalence using ELISA and PCR of HCMV among patient with congenital neural tube defect, hydrocephalus and microcephaly in Khartoum State. In the present study, HCMV IgM antibodies were found in 1(2%), and virus DNA was found in 4(8%) of the tested samples.

In Sudan, high seroprevalence of HCMV (IgM antibodies) among congenitally, infected neonates in Khartoum State (94.3%) was recorded by Nahla *et al.* (2011). This is in sharp contrast with the findings of present study. The difference between the two studies may be due to different patient inclusion criteria in the two studies depending on the type of specimens that were available at time of collection, hence in our study the specimens were from patients with tube defects, hydrocephaly and microcephaly, while in the other study specimen were mainly from cases of respiratory infection, hydrocephaly and jaundice.

On the other hand, Khairi *et al.* (2013) found CMV IgM antibodies in 6 % of pregnant women at Omdurman Maternity Hospital that was in concordance with the finding reported from Italy, 0.47% (Barbi M *et al.* 1998), Sweden, 0.46% (Ahlfors K *et al.* 1999) and US, 0.48% (Murph JR *et al.* 1998).

The discrepancy in the reported results of congenital CMV infection between ELISA test and conventional PCR could be due to differences in the sensitivity and specificity of the methods. The ideal diagnostic test should be sufficiently sensitive to detect infection at an early stage before clinically significant disease has occurred; the test should also eliminate the false positive results. Detection of HCMV DNA is most likely to be useful in this way because it has high sensitivity and more specificity than serological methods (ELISA). Positive IgM ELISA and negative DNA results might be to due to the persistence of the IgM antibodies for a long time after infection in some apparently healthy individual, where virus load may be too low to be detected by PCR. 

In conclusion, Cytomegalovirus showed low prevalence in infants with hydrocephalus, neural tube defect and microcephaly in Khartoum State. This study should pave the way for more research on various area of HCMV infection including different sources of virus, the association between HCMV acquisition and various risk factors such occupation, ethnicity, geographical location, hospitalization, antibiotic usage, surgery and disinfection. Also other causes of childhood congenital defects such as rubella virus should warrant further investigation.

## Figures and Tables

**Table 1:** cross- tabulation between PCR and ELISA (IgM) results for the diagnosis of HCMV in sera samples collected from neonates with congenital anomalies in Khartoum State, Sudan, during the period from September 2014 to January 2015.

ELISA	PCR		Total	Agreement
	Positive	Negative		
IgM Positive	1	0	1	94%
Negative	3	46	49	
Total	4	46	50	
